# Microbiota in Gingival Crevicular Fluid Before and After Mechanical Debridement With Antimicrobial Photodynamic Therapy in Peri-Implantitis

**DOI:** 10.3389/fcimb.2021.777627

**Published:** 2022-01-07

**Authors:** Haiyan Wang, Ye Liu, Wei Li, Wenyue Li, Hongtao Xu, Guangliang Niu, Zuomin Wang

**Affiliations:** ^1^ Department of Stomatology, Beijing Chaoyang Hospital, Capital Medical University, Beijing, China; ^2^ Peking University Fifth School of Clinical Medicine, Beijing, China; ^3^ The Key Laboratory of Geriatrics, Beijing Institute of Geriatrics, Institute of Geriatric Medicine, Chinese Academy of Medical Sciences, Beijing Hospital/National Center of Gerontology of National Health Commission, Beijing, China; ^4^ Department of Laboratory Medicine, Beijing Hospital, National Center of Gerontology, Institute of Geriatric Medicine, Chinese Academy of Medical Sciences, Beijing, China; ^5^ Department of Stomatology, Beijing Hospital of Integrated Traditional Chinese and Western Medicine, Beijing, China

**Keywords:** peri-implantitis, oral microbiota, photodynamic therapy, mechanical debridement therapy, 16S ribosomal RNA gene sequencing

## Abstract

**Objectives:**

This study aims to compare the microbiota of gingival crevicular fluid (GCF) before and after mechanical debridement (MD) with antimicrobial photodynamic therapy (aPDT) and determine the core efficient microbiota in peri-implantitis after treatment.

**Methods:**

We recruited 9 patients (14 implants) treated with MD+aPDT for peri-implantitis at our center from February 1, 2018, to February 1, 2019. GCF was collected using filter paper strip before and after the treatment. The bacterial 16S rRNA was amplified and sequenced using an Illumina MiSeq platform to characterize the GCF. Bioinformatics and statistical analyses were performed using QIIME2 and R.

**Results:**

A total of 4,110,861 high-quality sequences were obtained from GCF samples. Based on the reference database, 1,120 amplicon sequence variants (ASVs) were finally harvested. Principal coordinates analysis indicated significant differences in the bacterial community structure between the 180 days after-treatment group and pre-treatment group. Difference analysis and least discriminant analysis showed that the differences were mainly reflected in non-dominant bacteria between these two groups. The non-dominant genera with significantly different distribution between the 180 days after-treatment group and the pre-treatment group included *Lactobacillus*, *Pedobacter*, *Bulleidia*, *Centipeda*, *Desulfovibrio*, *Ochrobactrum*, *Staphylococcus*, *Microbacterium*, *Brevundimonas*, *Desulfobulbus*, and *Parvimonas*. Moreover, a total of 29 predictive functional categories at KEGG level 2 were identified. The significant difference pathways at KEGG level 2 between after-treatment and pre-treatment were concentrated in infectious disease-related pathways.

**Conclusions:**

Patients with peri-implantitis have significant changes in the low-abundance bacteria of the GCF before and after MD+aPDT. MD+aPDT may change the composition of GCF microbiota by increasing the abundance of cluster 1 (beneficial) and decreasing that of cluster 4 (harmful), which may decrease metabolic response to infection and thus improve peri-implantitis.

## Introduction

Implant restorations have gradually become one of the most important restoration methods in stomatology (Dragan et al., 2019). Peri-implantitis is one of the most common and difficult complications after placement of dental implants. The incidence of peri-implantitis is around 7.3%–28.3% (Song et al., 2020). As a frequent cause of peri-implantitis, bacterial infection can lead to a continuous inflammatory reaction that affects peri-implant hard and soft tissues. The most common manifestations of peri-implantitis include soft-tissue infections and bone loss around the implant, formation of a peri-implant pocket, and bleeding on probing (Lang et al., 2000; Prathapachandran and Suresh, 2012). Patients often suffer from redness, swelling, and pain around the implant, and the osseointegration around the implant will be damaged, which results in the loosening or eventual loss of the implant (Lindhe et al., 1992; Derks and Tomasi, 2015).

Studies (Sigusch et al., 2010; Froum et al., 2016; Malgikar et al., 2016; Sivaramakrishnan and Sridharan, 2018) have shown that mechanical debridement (MD) with antimicrobial photodynamic therapy (aPDT) (MD+aPDT) is more effective in the treatment of periodontal and peri-implant diseases compared with MD alone. aPDT involves the use of a non-toxic light-sensitive dye called a “photosensitizer” (PS) combined with harmless visible light (low energy) of appropriate wavelength to match the absorption spectrum of the PS (Dai et al., 2012). This procedure stimulates the dye to form free radicals of singlet oxygen that will act as toxic agents to the bacteria/cells (de Oliveira et al., 2007; Sculean et al., 2015). aPDT has also been reported to kill pathogenic microbes associated with the etiology of periodontal and peri-implant diseases, including *Aggregatibacter actinomycetemcomitans*, *Prevotella intermedia*, and *Porphyromonas gingivalis* (Javed and Romanos, 2013). The use of 16S ribosomal RNA (rRNA) gene sequencing has facilitated research on difficult-to-culture, low-biomass microbial communities present in the gingival crevicular fluid (GCF) (Aas et al., 2005). However, few studies have explored the change of oral flora before and after MD+aPDT in peri-implantitis. Here, in our current study, the 16S rRNA gene cloning library was amplified and constructed with polymerase chain reaction (PCR), and the gingival bacterial community was described for the implants after treatment at different time points, aiming to compare the microbiota over time in peri-implantitis treatment patients and evaluate the effectiveness of MD+aPDT.

## Results

### Basic Characteristics of Subjects and Reads

**Figure 1:**
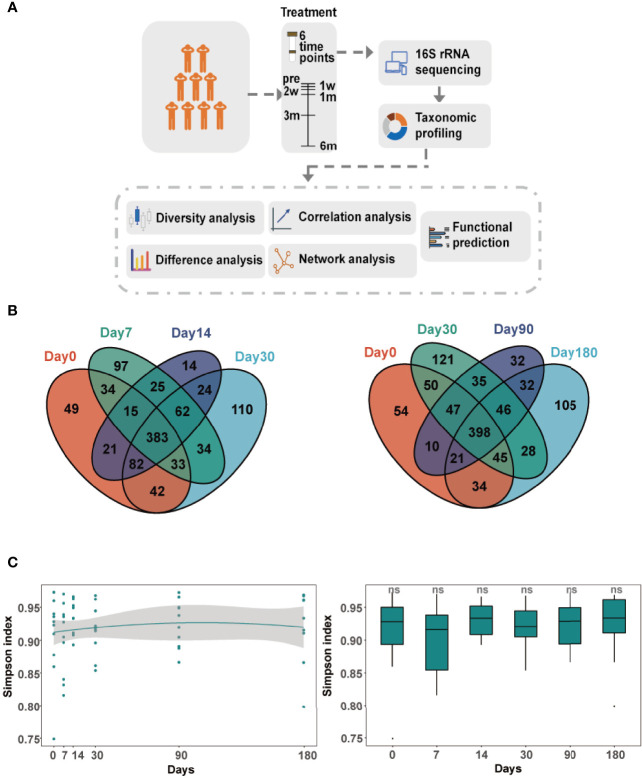
Longitudinal dynamics of gingival crevicular fluid (GCF) microbiota. **(A)** Overview of the study design and sample collection. **(B)** Venn diagram of amplicon sequence variants (ASVs) in six comparison groups. Day 0: before treatment; day 7: 7 days after treatment; day 14: 14 days after treatment; day 30: 30 days after treatment; day 90: 90 days after treatment; day 180: 180 days after treatment. **(C)** The alpha diversity of the GCF microbiota over time. The Simpson index compared between microbiota of each time point and before treatment. Significance was measured using the Wilcoxon rank-sum test (*P* > 0.05, ns).

We followed up patients with peri-implantitis at six time points (**Figure 1A**). A total of 61 samples were collected from nine patients. The severity of peri-implant inflammation was stage 3–4 by *Passi* classification. These patients included seven males (77.8%) and two females (22.2%), with an average age of 42.7 years. These 61 samples were obtained from 14 implants consisting of 10 molar implants (71.4%), 2 premolar implants (14.3%), and 2 anterior dental implants (14.3%) (**Additional File 1: Table S1**). The rarefaction curves of the number of reads plotted against the number of amplicon sequence variants (ASVs) indicated that the number of qualified reads from the used samples were adequate for the final analysis, as a further increase in the reads would have a minor effect on the obtained ASVs (**Additional File 6: Figure S1**). A total of 4,110,861 high-quality sequences were obtained from GCF samples. We finally detected 1,120 ASVs (**Figure 1B**).

### Diversity Analysis of the GCF Microbiota

**Figure 2:**
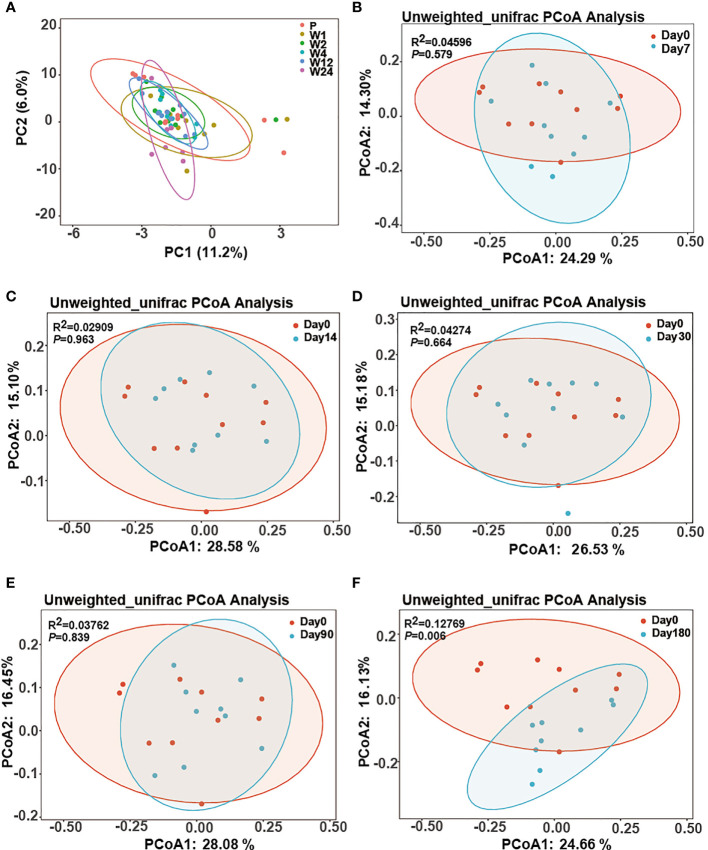
The beta diversity of the GCF microbiota over time. **(A)** PCA (unconstrained ordination) of all samples at the six time points. **(B–F)** Two-dimensional diagram of the principal coordinate analysis (PCoA) of unweighted UniFrac distance metrics of the ASV abundance data from the GCF sample of peri-implantitis patients across all time points. Each sample represented by the small circle and ellipses represents the 95% confidence interval.

The microbial diversity of GCF showed a little change over time after treatment. The alpha diversity analysis revealed that there were no significant differences in the GCF microbiota, which remained constant throughout the study (**Figure 1C**). The Bray–Curtis distance between microbiota of each time point and day 0 was also not significantly different (**Additional File 7: Figure S2**). The principal component analysis (PCA) clearly illustrated the dynamic process of GCF microbiota over time (**Figure 2A**). However, principal coordinates analysis (PCoA) using unweighted UniFrac distance showed GCF microbiota between different time points and day 0 (**Figures 2B–E**), with significant differences at day 0 and 180 days after treatment (PERMANOVA, *P*-value = 0.006, **Figure 2F**).

### Dynamic Change in Relative Abundances of Bacterial Taxa Over Time

**Figure 3:**
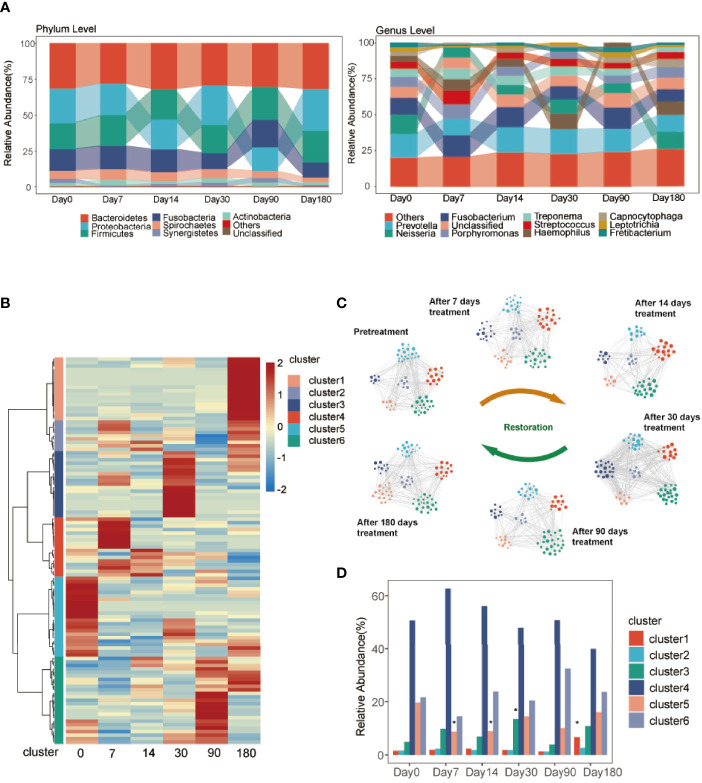
Taxonomies of GCF change over time. **(A)** Relative abundance of bacterial phyla and genera (top 10) over the six time points. **(B)** Hierarchical Ward-linkage clustering based on the Pearson correlation coefficients of the genera among six time points. The left panel shows hierarchical Ward-linkage clustering based on Pearson correlation coefficients of the genera, colored by the co-abundance groups (cluster). **(C)** The microbiota network of the clusters at the genus level. The size of the node represents the relative abundance of the genus. The color of the node represents the cluster identified in **(B)**. The edges are filtered with Spearman coefficients >0.7, *P < *0.05, FDR <10%. **(D)** The relative abundance of clusters at the six time points. Significance was measured using the Wilcoxon rank-sum test (*P* < 0.05, *).

The relative abundance of the ASVs at the phylum and genus levels changed over time (**Figure 3A**). At the phylum level, *Bacteroidetes*, *Proteobacteria*, *Firmicutes*, *Fusobacteria*, *Spirochaetes*, *Synergistetes*, and *Actinobacteria* were the dominant phyla. At the genus level, *Prevotella*, *Neisseria*, *Fusobacterium*, *Porphyromonas*, *Treponema*, *Streptococcus*, *Haemophilus*, *Capnocytophaga*, *Leptotrichia*, and *Fretibacterium* were the dominant genera.

To further explore the complexity in the developing GCF microbiota, we employed clustering analysis to ascertain GCF community types. All samples were grouped into six discrete clusters (**Figure 3B**) at the genus level (**Additional File 2: Table S2**). The relative abundance of the genera colored by clusters changed over time (**Figure 3C**). The significant differences in the relative abundance of clusters between after-treatment groups and day 0 are shown in **Figure 3D**. At 90 and 180 days, the relative abundance of cluster 6 was more than day 0 and that of clusters 4 and 5 was less than day 0. The relative abundance of cluster 1 between day 180 and day 0 was significantly different.

**Figure 4:**
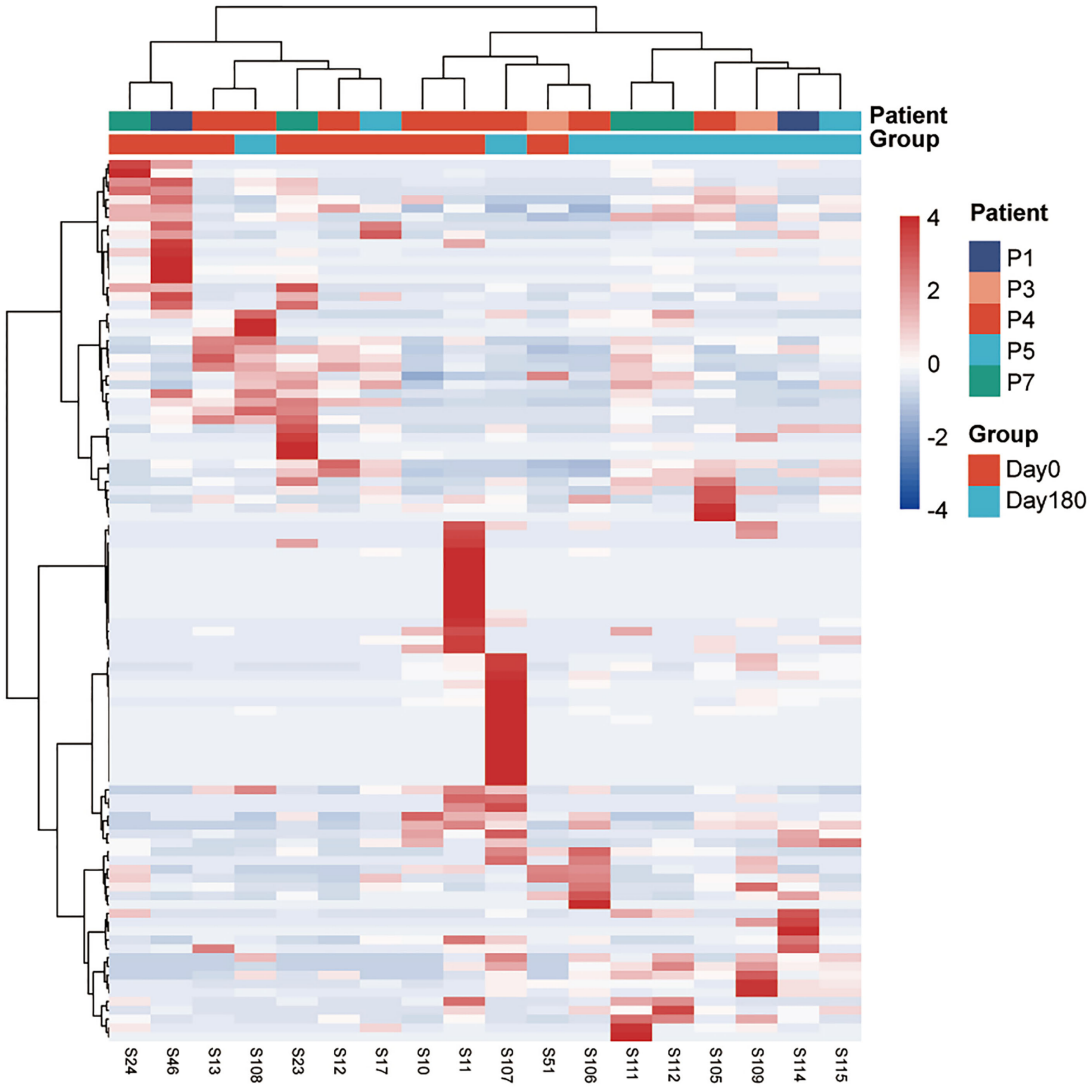
A heatmap of the relative abundances of ASVs between the 180 days after-treatment group and pre-treatment group (day 0). The top panel shows hierarchical Ward-linkage clustering based on Ward.D distance of all the 18 samples, colored by the groups and patients. The left panel shows hierarchical Ward-linkage clustering based on Pearson correlation coefficients of the genera.

**Figure 5:**
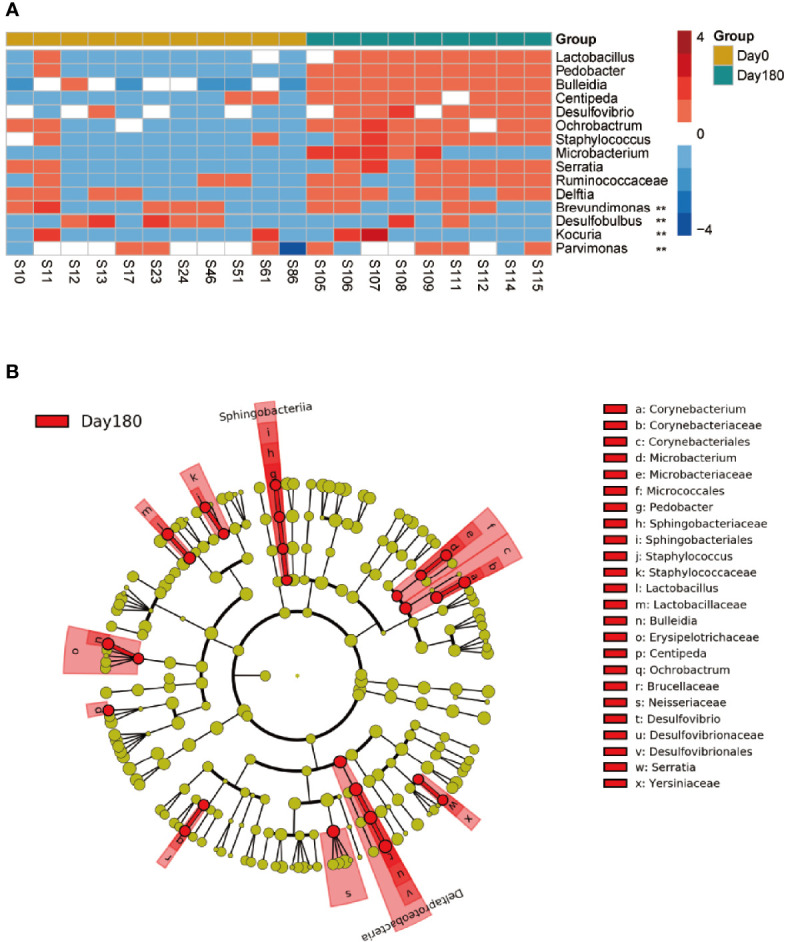
Different taxa between the pre-treatment group (day 0) and the 180 days after-treatment group. **(A)** Significantly different genera of day 0 compared with the 180 days after-treatment group. Wilcoxon rank-sum test with false discovery rate correction (*P* < 0.05, **). **(B)** Taxonomic LEfSe cladogram obtained using linear discriminant analysis (LDA) effect size (LEfSe) analysis. LEfSe identified the taxa with the greatest differences in abundance between the 180 days after-treatment group and day 0. Only taxa meeting a significant LDA threshold value of >2 are shown.

In addition, the heatmap showed significant difference in genera between day 0 and 180 days after treatment (**Figure 4**). The GCF microbiota from the same patients differed significantly after treatment. At the genus level, *Lactobacillus*, *Pedobacter*, *Bulleidia*, *Centipeda*, *Desulfovibrio*, *Ochrobactrum*, *Staphylococcus*, *Microbacterium*, *Brevundimonas*, *Desulfobulbus*, and *Parvimonas* were significantly higher 180 days after treatment (**Figure 5A**; **Additional File 3: Table S3**). To identify the specific communities associated with the treatment, LEfSe analysis revealed 26 discriminative features (LDA > 2, *P*-value < 0.05, **Figure 5B**; **Additional File 8: Figure S3**) at the class (*n* = 2), order (*n* = 4), family (*n* = 10), and genus (*n* = 10) levels. In the 180 days after-treatment group, the genera of discriminative features were *Lactobacillus*, *Ochrobactrum*, *Desulfovibrio*, *Staphylococcus*, *Pedobacter*, *Microbacterium*, and *Serratia*, which were grouped into cluster 1; other clusters included *Centipeda* (cluster 2), *Corynebacterium* (cluster 2), and *Bulleidia* (cluster 6).

### Pocket Probing Depth and Correlation With GCF Microbiota

**Figure 6:**
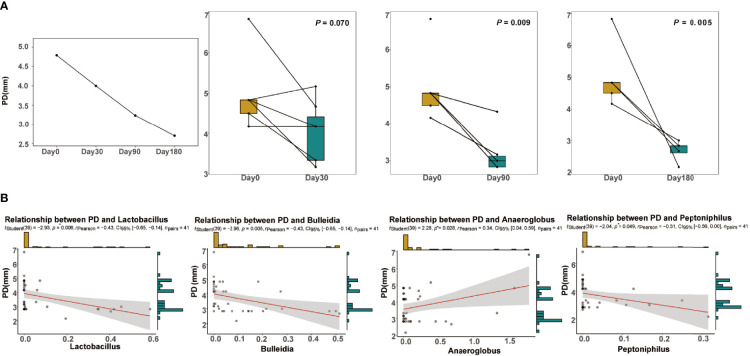
Clinical information. **(A)** Changes in the average probing depth (PD) over time at the four time points. Significance was measured using Wilcoxon rank-sum test between each time point and before treatment (*P* < 0.05). **(B)** The linear regression of PD against the relative abundance of the genera *Lactobacillus*, *Bulleidia*, *Anaeroglobus*, and *Peptoniphilus*. The 0.95 confidence interval is displayed.

Over time, the probing depth (PD) at 90 and 180 days was significantly decreased compared with that at day 0 (both *P* < 0.05; **Figure 6A**). To explore the interaction between clinical and microbial parameters related to peri-implantitis, Spearman’s correlation analysis was performed to estimate the relationships between PD and the genera (**Figure 6B**). The relative abundances of *Lactobacillus*, *Bulleidia*, and *Peptoniphilus* were negatively correlated with PD. The relative abundance of *Anaeroglobus* was positively correlated with PD. The relative abundances of *Lactobacillus*, *Bulleidia*, *Peptoniphilus*, and *Anaeroglobus* changed over time (**Additional File 9: Figure S4**).

### Functional Annotation of the Microbes of the GCF

**Figure 7:**
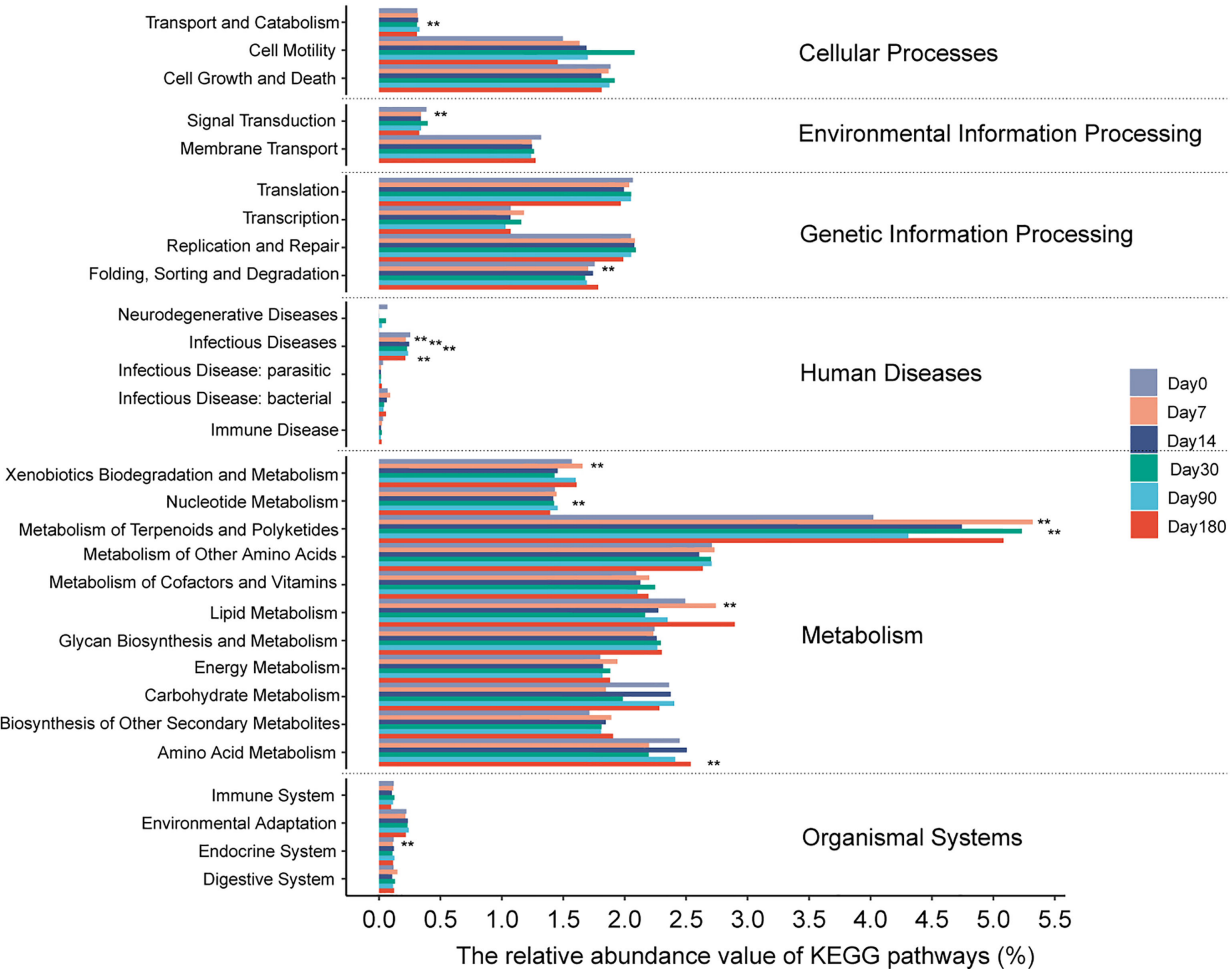
The relative abundance of each predicted functional category given in the KEGG pathways (level 2). The functional pathway between each time point and before treatment. Significance was measured using the Wilcoxon rank-sum test (*P* < 0.05, **). Day 0: before treatment; day 7: 7 days after treatment; day 14: 14 days after treatment; day 30: 30 days after treatment; day 90: 90 days after treatment; day 180: 180 days after treatment.

A total of 29 predictive categories in the KEGG level 2 functional modules were identified in the microbes of the GCF (**Figure 7**). Metabolism, genetic information processing, and cellular processes were the modules where the bacterial functions were concentrated. The significant difference pathways in the KEGG level 2 between after-treatment time points and day 0 were concentrated in infectious disease-related pathways (**Additional File 4: Table S4**). The mean proportions of the infectious disease-related pathways in the after-treatment groups were all less than those in the pre-treatment group. All the pathways at KEGG level 3 showing significant difference between after treatment and day 0 are shown in **Additional File 10: Figure S5** and **Additional File 5: Table S5**.

## Discussion

In this prospective cohort study, we used a comprehensive, multidimensional approach to investigate the GCF microbiota in peri-implantitis after treatment at six time points. This might be beneficial and indicative for future studies focusing on the dynamic change of oral microbiota after peri-implantitis therapy.

In the present study, the dominant genera in all subjects included *Prevotella*, *Neisseria*, *Fusobacterium*, *Porphyromonas*, *Treponema*, *Streptococcus*, *Haemophilus*, *Capnocytophaga*, *Leptotrichia*, and *Fretibacterium*, which were in line with previous findings indicating the red and orange complex genera in peri-implantitis (Socransky et al., 1998; Li et al., 2015). Although not statistically significant in alpha diversity, PCoA analysis showed a significantly different bacterial community structure between the pre-treatment group and the 180 days after-treatment group.

It has been reported (Jovanovic, 1993; Kumar et al., 2012; Maruyama et al., 2014) that *Prevotella*, *Treponema*, *Campylobacter*, and *Porphyromonas* were pathogenic bacteria genera in peri-implantitis. However, analysis of species abundance suggested no significantly different distribution at the genus levels, involving the dominant bacteria, which was not in line with previous findings. In addition, the difference analysis and least discriminant analysis showed that the differences were mainly reflected in non-dominant bacteria between the pre-treatment group and the 180 days after-treatment group. Unlike many human diseases, oral bacterial diseases, such as caries and periodontitis, are not caused by a single species but by a consortium of species that are likely living harmlessly in very low numbers (often below the limit of detection) in the oral cavity (Krishnan et al., 2017). Therefore, we divided the genera into six clusters. At 90 and 180 days, the relative abundance of cluster 4 was less than day 0, and the relative abundance of cluster 1 between day 180 and day 0 was significantly different. We found that peri-implantitis pathogen genera such as *P. gingivalis*, *Tannerella forsythia*, and *P. intermedia* were among the members of cluster 4. Furthermore, *Lactobacillus*, a beneficial bacteria, was among the members of cluster 1. MD+aPDT might play a role in changing the composition of GCF microbiota by increasing the abundance of cluster 1 and decreasing cluster 4 to improve peri-implantitis. The “keystone pathogen” hypothesis suggests that pathogens of low abundance can cause inflammatory diseases by rendering a symbiotic microbial community dysbiotic (Hajishengallis et al., 2012). Based on network analysis, it is possible that low-abundance genera play key roles and contribute to an overall shift in the microbial community. The correlation analysis showed that *Lactobacillus*, *Bulleidia*, and *Peptoniphilus* increased, and *Anaeroglobus* decreased, which might improve peri-implantitis, although the correlation was not strong.

The KEGG pathway was significantly concentrated in infectious diseases between the different time points and day 0, which could explain the role of MD+aPDT in shifting the microbiota to decrease metabolite-related infections then improve peri-implantitis. Metabolism, genetic information processing, and cellular processes were the most abundant pathways among the 29 matched KEGG pathways, supporting the conventional function of microorganisms that involved the communication between the hosts and bacteria. As suggested by our current study, the differences in metabolism were mainly caused by non-dominant bacteria; however, due to the limitations of 16S sequencing function prediction, more research in metagenomics is warranted.

Although our findings could not directly prove that MD+aPDT can dramatically decrease the main pathogens in patients with peri-implantitis, it can be speculated that this treatment strategy can alter the low-abundance bacterial community.

Our current study was limited by its small sample size and the absence of healthy implant controls. In addition, many other influential factors (diet, lifestyle, and oral care) of the GCF microbiota were not investigated. Nonetheless, to our knowledge, this is the first study to compare the GCF microbiota before and after MD+aPDT in peri-implantitis.

In conclusion, patients with peri-implantitis have significant changes in the low-abundance bacteria of the GCF before and after MD+aPDT. Our results showed that there were significant differences in the diversity of subgingival bacteria between the 180 days after-treatment group and day 0 group. MD+aPDT may change the composition of GCF microbiota by increasing the abundance of cluster 1 (beneficial) and decreasing that of cluster 4 (harmful), which may decrease metabolic response to infection and thus improve peri-implantitis. These findings may improve our understanding of the dynamic change of oral microbiota after peri-implantitis therapy.

## Materials and Methods

### Ethics and Informed Consent

This study was approved by the Ethics Committee of Beijing Chaoyang Hospital affiliated to Capital Medical University (approval number: 2018-Section-9). All patients participating in the study signed the informed consent form before the trial began.

### Study Subjects

This prospective cohort study recruited nine patients with dental implants with inflammation from the Department of Stomatology of Beijing Chaoyang Hospital affiliated to Capital Medical University from February 1, 2018, to February 1, 2019. A total of 77 samples of GCF were obtained from these 9 patients (14 implants), among which 61 samples succeeded in library construction and 16 failed. The inclusion criteria of the patients were as follows: a) having undergone dental implantation, b) with at least one dental implant for more than half a year, c) with a diagnosis of peri-implantitis (not only the first time), and d) understood the study and signed the informed consent form. The exclusion criteria were as follows: a) with a systemic disease, b) having taken immunosuppressive agents or antibiotics in the past 3 months, c) long-term use of contraceptive drugs, d) pregnant women, and e) smokers.

For implants suspected of having peri-implantitis, the mesial buccal, buccal, distal buccal, mesial lingual, lingual, and distal lingual surfaces were investigated. The main measures included periodontal probing depth (PD), plaque index (PLI) around the implant, sulcular bleeding index (SBI), and purulent conditions. Cone-beam computed tomography (CBCT) was performed in each patient to detect bone attachment. The diagnostic criteria of peri-implantitis were as follows: a) obvious inflammatory symptoms around the implant, b) bone loss revealed by X-ray examination, c) possible hemorrhage and suppuration, d) at least one implant site with periodontal PD ≥6 mm, e) PLI around the implant ≥2 points, and f) visible bleeding around the implant after probing, with an SBI of ≥2 points.

Patients with peri-implantitis were treated with MD+aPDT. The specific therapy method was as follows: MD used the glycine subgingival sandblasting method and removed plaque around the implant. MD treatment time was 5 s per site. The APDT machine (Helbo Photodynamic Systems, Wels, Germany) and methylene blue were used after MD. The wavelength of the photodynamic system diode light source was 635 nm. The range of 3D illumination was a minimum of 60 mW/cm². APDT time was 10 s per site. GCF samples were taken before treatment and 7, 14, 30, 90, and 180 days after treatment for amplicon sequencing and analysis. The workflow is shown in **Figure 1A**.

### Sample Collection and DNA Extraction

GCF samples were collected using filter paper strip. First, the plaque on the surface of the implant was removed, followed by the insertion of the Whatman I periodontal test strip (2 mm × 10 mm) into the gingival sulcus. There were a total of four inserting sites for each implant, that is, the proximal and distal points of both the buccal and tongue sides. Insertion was stopped when there was a feeling of slight resistance, and the insert was removed after 30 s. If the filter paper was contaminated with blood stains, it was discarded and retested after hemostasis. The GCF amount of each test implant was the sum of the above four sites. The wet parts of the four filter papers of each test implant were cut into an Eppendorf tube, sealed with tin foil, and quickly stored at −80°C for further testing.

One milliliter of sterile distilled water was added to the Eppendorf tube, shaken for 1 min, and mixed well, and then the filter paper strips were removed. The samples were centrifuged at 12,000 rpm for 5 min and the bacteria were pelleted. Deoxyribonucleic acid (DNA) extraction was performed by using the Mora-Extract DNA extraction kit (Tokyo, Kyokuto Pharmaceutical, Japan) according to the instructions of the manufacturer. Two hundred microliters of Tris-EDTA (ethylenediaminetetraacetic acid) was used as the elution buffer and the mixture was stored at −20°C before use.

### Polymerase Chain Reaction Amplification and 16S rRNA Cloning Library Construction

With these DNA templates, PCR was performed on the real-time PCR machine (Roche, Basel, Switzerland) with barcode-specific primers. The bacterial diversity identification corresponding regions included the 16S V3–V4 regions (primers 515F: 5′-GTGCCAGCMGCCGCGGTAA-3′ and 806R: 5′-GGACTACHVGGGTWTCTAAT-3′). The 16S rRNA gene was amplified in the V3–V4 regions using the following 50-μl reaction system: 10× polymerase buffer, 2.5 mM dNTPs, 0.2 μM primer, 1.25 U Takara Ex TaqHot Start reagent (TaKaRa Biomedicals, Tokyo, Japan), and 1–2 μl of template DNA. The PCR cycle condition included an initial denaturation at 94°C for 1 min; 35 cycles of 98°C for 10 s, 55°C for 30 s, and 72°C for 2 min; and a final extension at 72°C for 1 min. After electrophoresis, the PCR products were purified with the AMPure paramagnetic beads kit (Agencourt Bioscience Corporation, MA, USA). The dual-indexed amplicon mixture was pooled according to the instructions of the manufacturer (Illumina, Inc., San Diego, CA, USA), and then sequenced on the Illumina MiSeq platform to produce 2 × 250 base pair reads.

### Microbiota Analysis

Illumina reads were trimmed to remove bases that had a PHRED score of <25 using BGI quality-filtering pipeline. Quality trimmed reads were then demultiplexed and paired-end reads were joined both using Quantitative Insights into Microbial Ecology 2 software (QIIME2, Version 2019.10) (Bolyen et al., 2019) with default settings. The merged reads were processed through the Deblur to reduce noise, filter chimeric reads, and obtain the amplicon sequence variant (ASV) feature sequences. Taxonomies were assigned using the Human Oral Microbiota Database (HOMD, Version 15.1) as the reference database.

Alpha diversity (such as observed ASVs, Chao1, Shannon, and Simpson) and beta diversity measures were analyzed using the R software (Version 3.6.1), and the Wilcoxon and Kruskal–Wallis tests were used for two- and multigroup comparisons, respectively. Principal coordinate analysis (PCoA) was used to interrogate the robustness of group-wise clustering, and Adonis test was used to estimate group-wise beta diversity. Differential abundance analysis of the genera was carried out using R and *P*-values were adjusted for multiple testing (FDR-adjusted Wald test). Hierarchical Ward-linkage clustering was based on Pearson correlation coefficients of the genera, identifying different clusters by the co-abundance groups. The co-occurrence network of the genera colored by clusters was calculated using Spearman correlation coefficients. The thresholds of Spearman correlations were *r >*0.7 and *P*-value ≤0.05. Biomarker discovery analysis was performed using the LEfSe tool. Linear discriminant analysis (LDA) scores greater than 2.0 were considered to be significant. Correlations between clinical parameters PD and bacterial genera were also calculated using Spearman correlation coefficients (*P*-value ≤ 0.05). Microbial functions were predicted by PICRUSt2 (Phylogenetic Investigation of Communities by Reconstruction of Unobserved States) based on high-quality sequences (Langille et al., 2013) (KEGG PATHWAY Database http://www.genome.jp/kegg/pathway.html). A *P*-value or *q*-value ≤0.05 was considered as statistically significant.

## Data Availability Statement

The datasets presented in this study can be found in online repositories. The names of the repository/repositories and accession number(s) can be found below: NCBI PRJNA786326.

## Ethics Statement

The studies involving human participants were reviewed and approved by the Ethics Committee of Beijing Chaoyang Hospital affiliated to Capital Medical University (approval number: 2018-Section-9). The patients/participants provided their written informed consent to participate in this study.

## Author Contributions

ZW conceived and designed the experiments. HW and YL conducted the experiments and data analysis. WYL and WL performed the collection of clinical samples and case information. HW, WYL, and WL performed the sample pre-treatment. HW and YL wrote the manuscript. ZW, HX, and NG played advisory roles. All authors contributed to the article and approved the submitted version.

## Funding

This work was supported by the National Key Research and Development Program of China (Grant 2018YFC2000505) and the Applied Research Program of Capital Clinical Features (Grant Z18110001718172).

## Conflict of Interest

The authors declare that the research was conducted in the absence of any commercial or financial relationships that could be construed as a potential conflict of interest.

## Publisher’s Note

All claims expressed in this article are solely those of the authors and do not necessarily represent those of their affiliated organizations, or those of the publisher, the editors and the reviewers. Any product that may be evaluated in this article, or claim that may be made by its manufacturer, is not guaranteed or endorsed by the publisher.

## Supplementary Material

Supplementary Table S1 Sample information.

Supplementary Table S2 Genera in co-abundance group used for network construction.

Supplementary Table S3 Genera with statistic significant between pre-treatment and 180 days after treatment.

Supplementary Table S4 The significant difference pathways in the KEGG level 2 between different time points.

Supplementary Table S5 The significant difference pathways in the KEGG level 3 between different time points.

Supplementary Figure S1 Rarefaction curves of the numbers of reads from the GCF samples of 6 time points.

Supplementary Figure S2 The beta diversity of the GCF microbiota over time. The bray-curtis distance compared between microbiota of each time point and pre. Significance was measured using Wilcoxon rank-sum test (*P*>0.05, ns).

Supplementary Figure S3 Taxonomic LEfSe plot obtained using linear discriminant analysis (LDA) effect size (LEfSe) analysis. LEfSe identified the taxa with the greatest differences in abundance between the 180 days after-treatment group and pre-treatment group. Only taxa meeting a significant LDA threshold value of >2 are shown.

Supplementary Figure S4 The relative abundance of *Lactobacillus*, *Bulleidia*, *Peptoniphilus*, and *Anaeroglobus* over time.

Supplementary Figure S5 The relative abundance of each predicted functional category given in the KEGG pathways (level 3). The functional pathway compared between each time point and pre. Significance was measured using Wilcoxon rank-sum test (*P*<0.05, **). Only statistic significant pathways are shown. Day0: before treatment; Day7: 7 days after treatment; Day14: 14 days after treatment; Day30: 30 days after treatment; Day90: 90 days after treatment; Day180: 180 days after treatment.
