# Supplementary material for: Microbiota in Gingival Crevicular Fluid Before and After Mechanical Debridement With Antimicrobial Photodynamic Therapy in Peri-Implantitis
Source: Front Cell Infect Microbiol. 2022 Jan 7;11:777627. doi: 10.3389/fcimb.2021.777627 (PMC8791307; doi:10.3389/fcimb.2021.777627)
Supplement: Supplementary Table S1 — Sample information. [file Table_1.docx]

Additional file 1: Table S1. Sample information.

| Subject | Sex | Age | Teeth site | Sample ID | | | | | |
| --- | --- | --- | --- | --- | --- | --- | --- | --- | --- |
|  |  |  |  | Day0 | Day7 | Day14 | Day30 | Day90 | Day180 |
| P1 | Male | 41 | 35 | 46 | 47 | 49 | 55 | / | 114 |
| P2 | Male | 35 | 36 | / | 48 | / | / | / | / |
| P3 | Female | 32 | 12 | 51 | / | / | 63 | 95 | 109 |
| P4 | Male | 54 | 36 | 10 | 20 | / | / | 75 | 107 |
|  |  |  | 26 | 11 | 19 | 32 | 42 | 74 | 106 |
|  |  |  | 14 | 12 | 22 | 33 | 45 | 73 | 105 |
|  |  |  | 37 | 13 | 21 | 30 | 43 | 76 | 108 |
| P5 | Male | 23 | 11 | 17 | 27 | 34 | 54 | 83 | 115 |
| P6 | Male | 47 | 37 | / | 6 | 14 | 39 | 80 | / |
|  |  |  | 26 | / | 7 | 15 | 38 | 79 | / |
| P7 | Female | 49 | 46 | 23 | / | 36 | 57 | 82 | 112 |
|  |  |  | 26 | 24 | / | 35 | 56 | 81 | 111 |
| P8 | Male | 47 | 46 | 86 | / | / | / | / | / |
| P9 | Male | 56 | 47 | 61 | 66 | 71 | 77 | ~~/~~ | / |
