# Supplementary figures and images for: Microbiota in Gingival Crevicular Fluid Before and After Mechanical Debridement With Antimicrobial Photodynamic Therapy in Peri-Implantitis
Source: Front Cell Infect Microbiol. 2022 Jan 7;11:777627. doi: 10.3389/fcimb.2021.777627 (PMC8791307; doi:10.3389/fcimb.2021.777627)

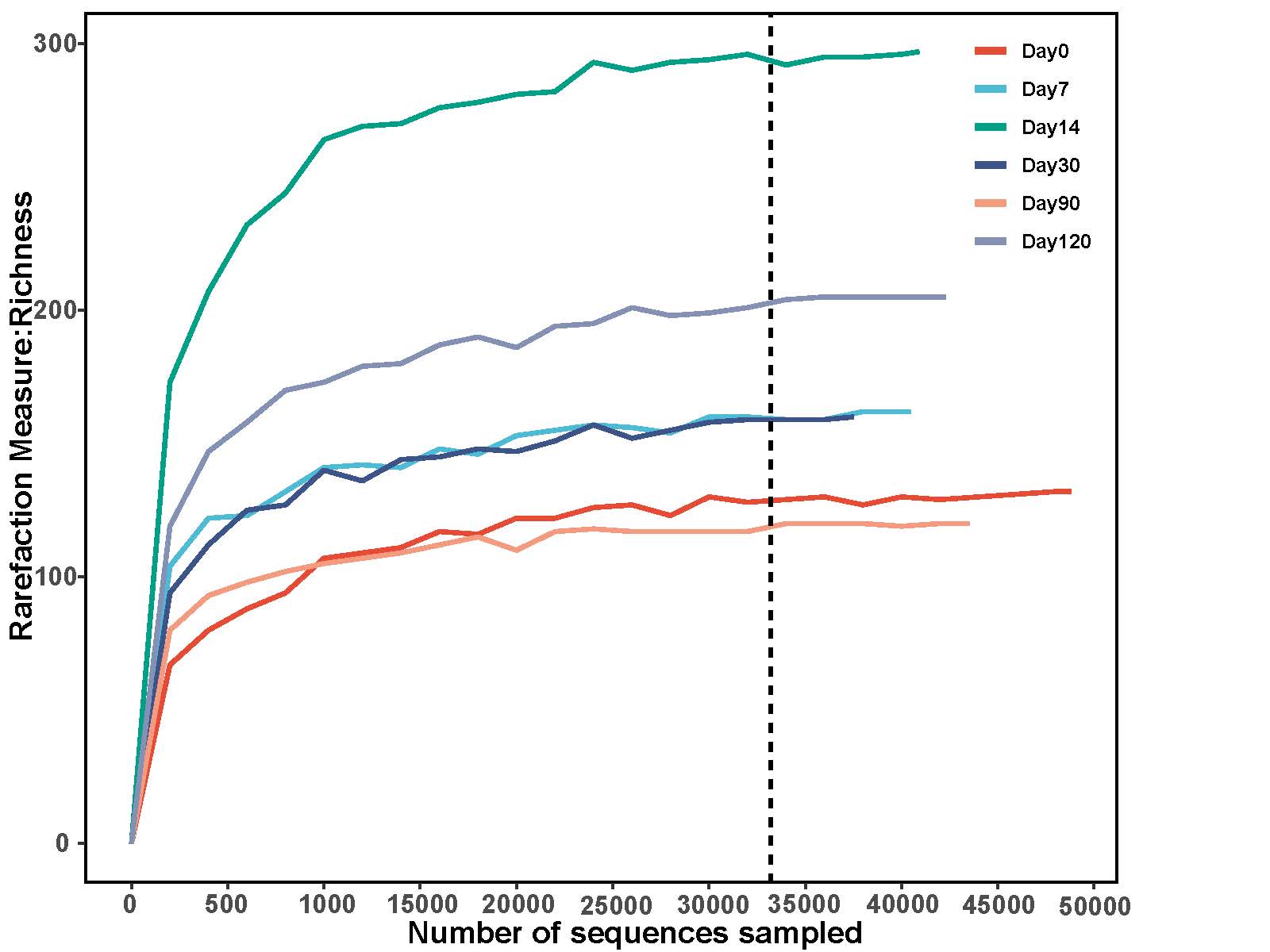

Supplement: Supplementary Figure S1 — Rarefaction curves of the numbers of reads from the GCF samples of 6 time points. [file Image_1.jpeg]

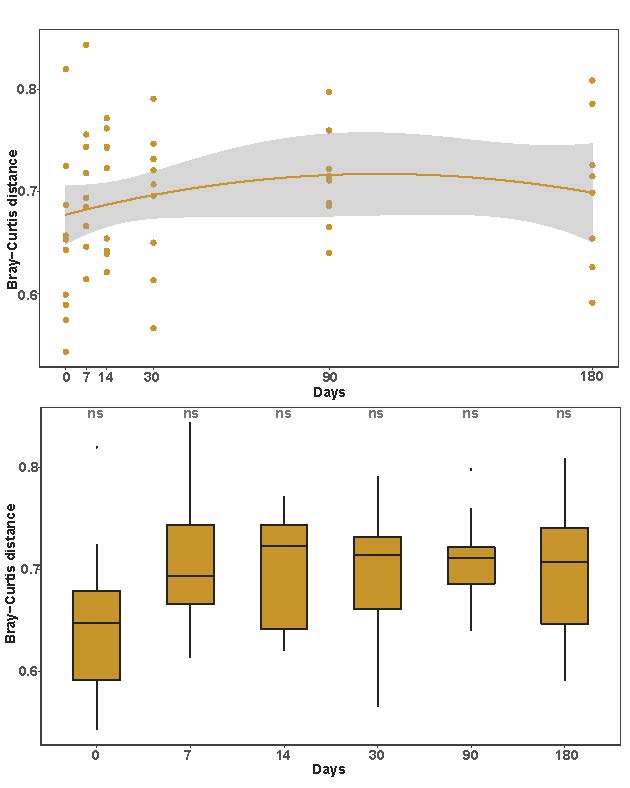

Supplement: Supplementary Figure S2 — The beta diversity of the GCF microbiota over time. The bray-curtis distance compared between microbiota of each time point and pre. Significance was measured using Wilcoxon rank-sum test (P>0.05, ns). [file Image_2.jpeg]

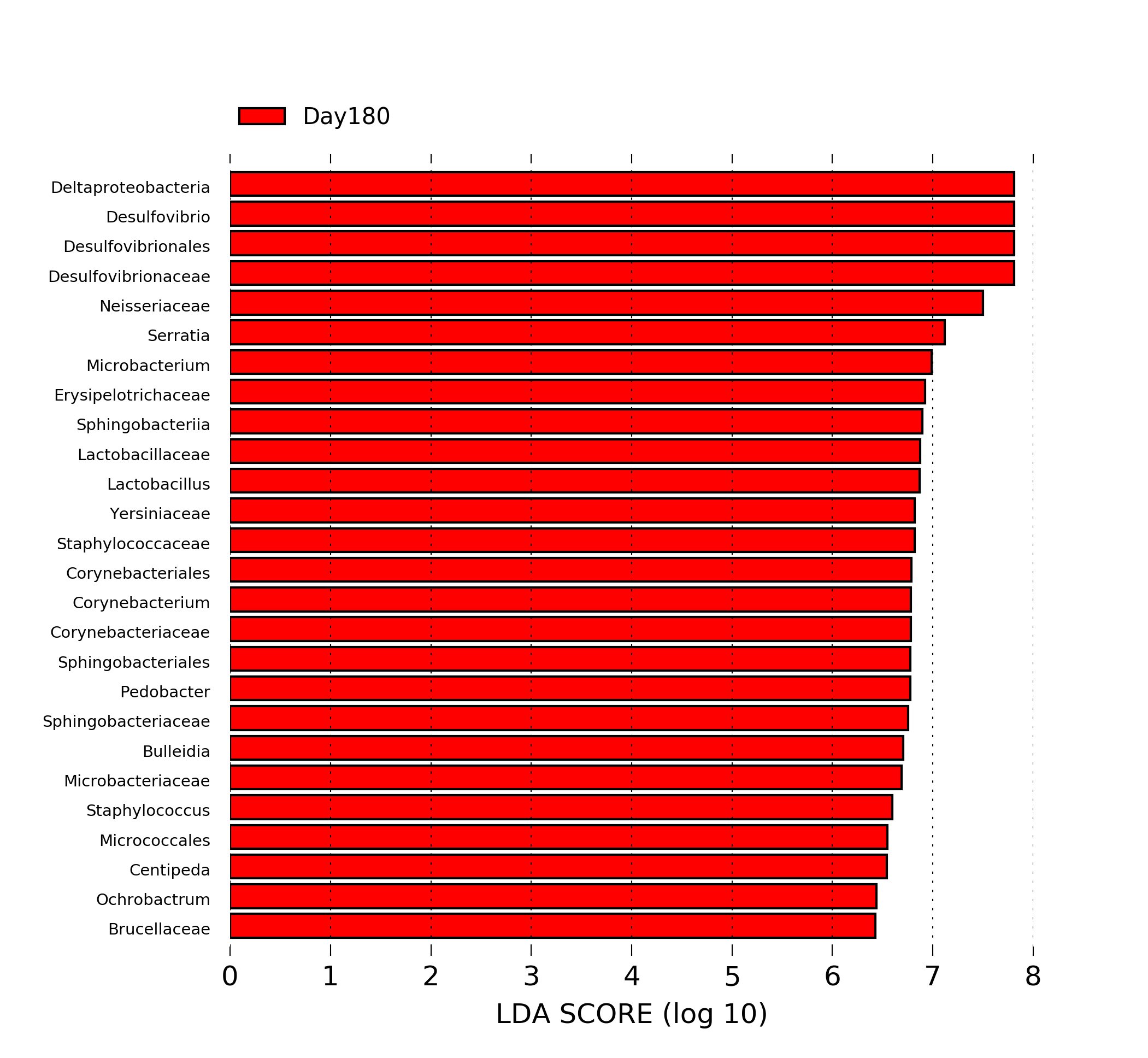

Supplement: Supplementary Figure S3 — Taxonomic LEfSe plot obtained using linear discriminant analysis (LDA) effect size (LEfSe) analysis. LEfSe identified the taxa with the greatest differences in abundance between the 180 days after-treatment group and pre-treatment group. Only taxa meeting a significant LDA threshold value of >2 are shown. [file Image_3.jpeg]

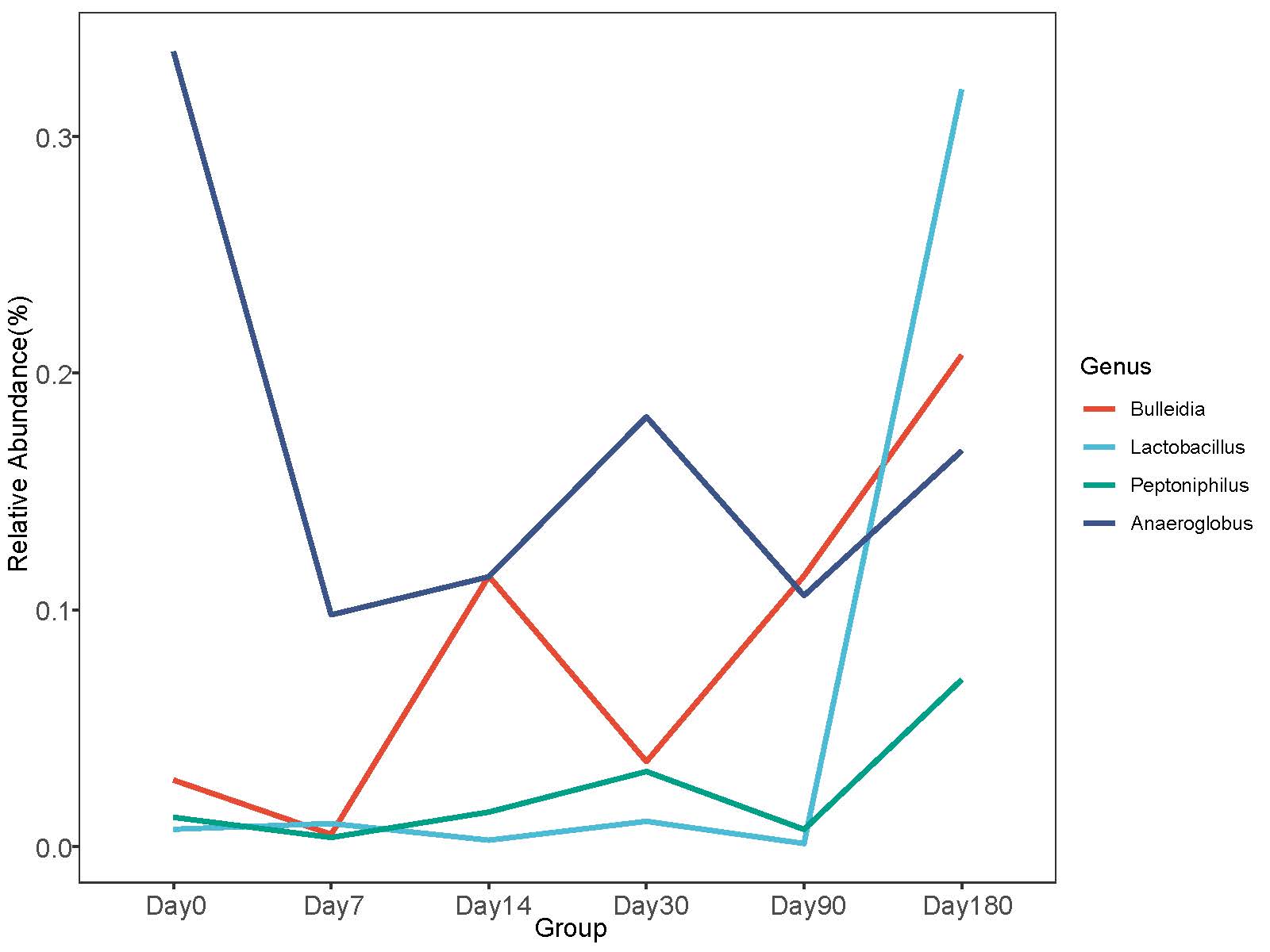

Supplement: Supplementary Figure S4 — The relative abundance of Lactobacillus, Bulleidia, Peptoniphilus, and Anaeroglobus over time. [file Image_4.jpeg]

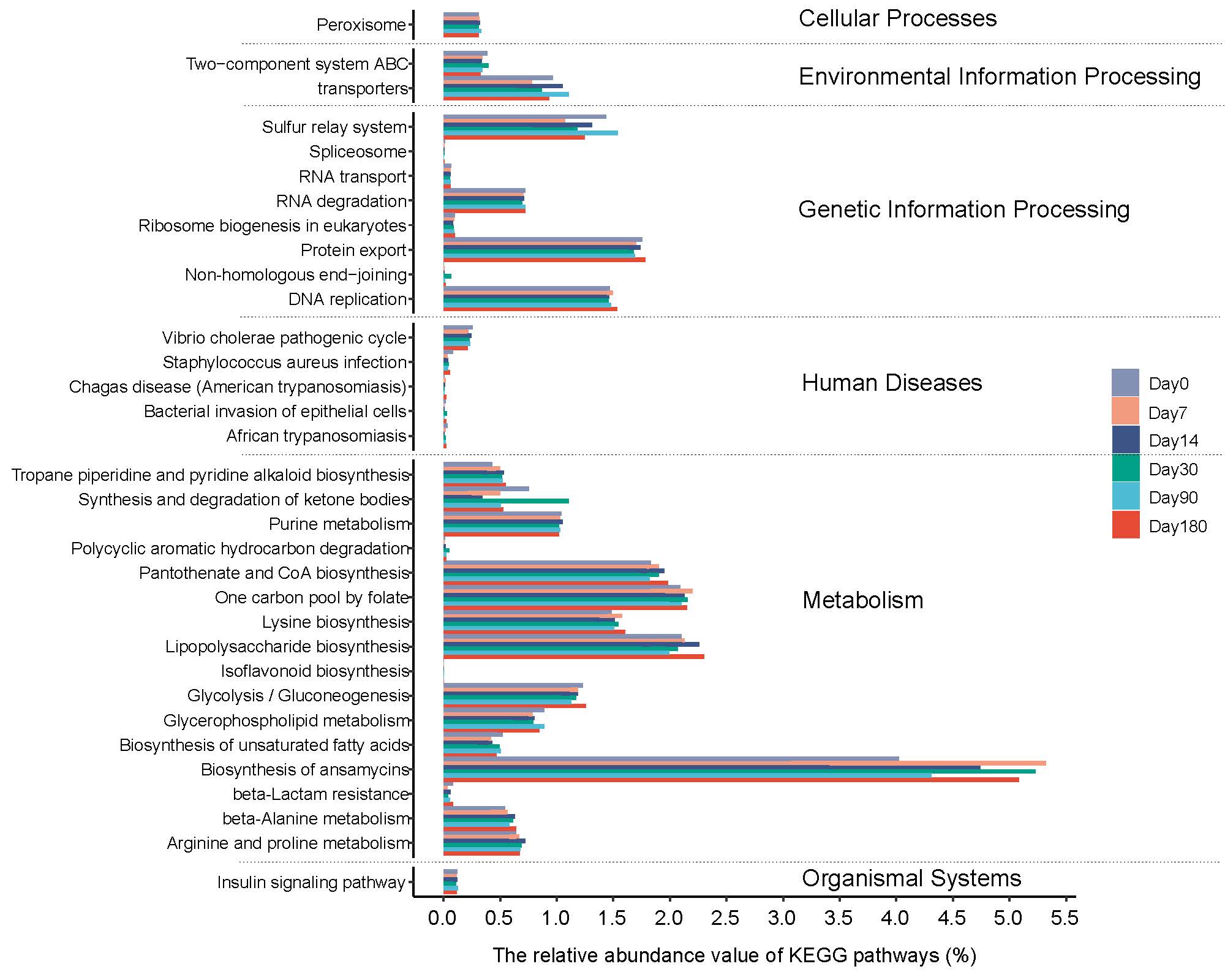

Supplement: Supplementary Figure S5 — The relative abundance of each predicted functional category given in the KEGG pathways (level 3). The functional pathway compared between each time point and pre. Significance was measured using Wilcoxon rank-sum test (P<0.05, **). Only statistic significant pathways are shown. Day0: before treatment; Day7: 7 days after treatment; Day14: 14 days after treatment; Day30: 30 days after treatment; Day90: 90 days after treatment; Day180: 180 days after treatment. [file Image_5.jpeg]
